# A prospective pilot cohort analysis of crash characteristics and pattern of injuries in riders and pillion passengers involved in motorcycle crashes in an urban area in Cameroon: lessons for prevention

**DOI:** 10.1186/s12889-015-2290-4

**Published:** 2015-09-18

**Authors:** Alain Chichom-Mefire, Julius Atashili, Jean G. Tsiagadigui, Clovis Fon-Awah, Marcelin Ngowe-Ngowe

**Affiliations:** Faculty of Health Sciences, University of Buea and Regional Hospital Limbe, P.O. Box 25526, Yaoundé, Cameroon; Faculty of Health Sciences, University of Buea, Buea, Cameroon; Surgical Unit, Laquitinie Hospital, Douala, Cameroon

**Keywords:** Traffic related injuries, Motorcycle, Riders, Pillion passenger, Pattern of lesions, Severity, Outcome

## Abstract

**Background:**

Low and middle-income countries carry over ninety per cent of the burden of injury related mortality and disability. Motorcycles are gradually becoming a major mode of transportation in Cameroon and other African countries in the absence of an organized public transport. Consequently, the contribution of motorcycle crash to injury-related deaths seems to be on the rise. Currently, data addressing motorcycle crash characteristics, pattern, and severity of motorcycle-related injuries in Cameroon are scarce. We hypothesised that head and limb injuries are the most frequent cause of morbidity and mortality and equally affect riders and pillion passengers.

**Methods:**

This hospital-based prospective pilot cohort analysis involving 405 motorcycle crashes and 621 injury victims was conducted in Laquintinie Hospital, a large centre located in an urban area in Cameroon. All motorcycle riders and passengers received in the emergency department over a 4 months period with an injury following a traffic related crash were included. Crash characteristics and type, anatomical location and severity of injuries were recorded and analysed comparing the pattern of injuries between riders and pillion passengers involved in motorcyclecrashes. This pilot analysis is expected to propose a snapshot of motorcycle injuries in Douala and will be followed by a larger analysis over a longer period.

**Results:**

We recorded a majority of motorcycle versus car and motorcycle versus motorcycle collisions. Most of these crashes occurred over the week-end and in the night. Helmet use was almost inexistent. We observed that females aged above 40 years represented the majority of pillion passengers. This accounted for the sex-ratio of 1.1/1. A total of 1311 injuries were identified in our patients, giving a mean of 2.1 injuries per victim. The head and the limbs were the most affected anatomical areas. Riders carried a higher risk of sustaining an injury to head and neck than pillion passengers. Riders and pillion passengers carried equal risk of injury to the lower limbs. Emergency room mortality was 4 · 3 % and riders were more likely to die than pillion passengers.

**Conclusion:**

This study has identified females aged above 40 years as a special vulnerable group in Douala. It also carries strong messages useful for the implementation of preventive measures and management of patients injured in motorcycle-related crash in general.

## Background

The global burden of injuries is globally on the rise [1–3]. Injuries will likely become the fourth leading cause of mortality and disability worldwide by 2030 [4]. Low and middle income countries (LMICs) are disproportionately affected, accounting for over 90 % of the overall injury related mortality [3]. Most of these deaths are attributable to motor vehicle crash. According to World Health Organization (WHO), in 2002 traffic related injuries killed an estimated 1.2 million people and injured more than 50 million others [5]. In African countries, it has been estimated that the number of people who die in traffic related injuries will undergo a 144 % increase between 1990 and 2020 [6].

Current data on injuries have permitted the identification of vulnerable road users. It appears that pedestrians and motorised two-wheeled vehicle users get injured on the road more frequently and more severely than other road users [7–15]. Analysis of motor vehicle crash related deaths indicates that over 40 % of victims are motorised two-wheeled vehicle users [10, 14].

Despite these established facts, motorcycle use as a mean of transportation is on the rise worldwide [7, 15, 16]. In Sub-Saharan African countries, the motorcycle and motorized two-wheelers have emerged as a popular commercial mode of transportation without any corresponding improvement and adaptation of road infrastructures [8, 17, 18]. This is particularly true for Cameroon where road infrastructures are generally considered very poor. It has been estimated that the number of people who die as a result of traffic injuries in this country is 35 times higher than what has been reported on similar road infrastructures in the United States of America and usually concerns vulnerable road users [19]. One third of traffic injury victims in large cities of Cameroon are motorcyclists [20]. Commercial motorcyclists popularly known in Douala as “Bend Skin” are a very frequently used a mode of transportation in the absence of any regular organized public transport. “Bend Skins” have become a prominent feature displayed on the roads in Douala. The motorcycle operators (riders) generally lack training, operate without a driving licence and the rules about helmet use and drunk driving which exist in Cameroon are generally not implemented.

The profile of motorcycle crash victims and other factors determining nature and severity of injuries have been studied, especially in developed countries [13, 15, 21, 22]. Many recent reports indicate that motorcycle accident victims are young males [11, 16, 21, 23] who display a unique pattern of injuries with a greater vulnerability of head and lower limbs [11, 16, 21, 24]. Few reports have shown interest in the differential analysis of injuries and their severity between riders and passengers [16, 21, 24].

While in developed countries, effective intervention plans are implemented with measurable results [25–27], low and middle income countries still lack the most basic epidemiological data.

In Cameroon, no study has specifically addressed the problem of motorcycle related injuries. Without such data, no reasonable and efficient policy of control of these injuries can be implemented.

The aim of this study was to describe the crash characteristics and epidemiological profile of motorcycle-related injury victims in a large hospital in Douala, Cameroon. We also analysed the pattern of injuries and in-hospital outcome comparing riders and pillion passengers. We hypothesized that most victims of motorcycle related crash are injured in a car versus motorcycle collision, that males below 40 years represent the large majority of cases and that the head and lower limb are the most affected body areas for both riders and passengers.

## Methods

### Study design and settings

This prospective cohort study was conducted in the emergency department of Laquintinie Hospital located in Douala, Cameroon over a four months period, from January 1^st^ to April 30^th^ 2012. Douala is the largest city of Cameroon with a total population estimated to be over 2.3 million inhabitants in 2010.

Laquintinie hospital is the largest referral centre in the country with a total capacity of over 450 beds. It possesses most specialized services, including an emergency department which is functional round the clock. The hospital is opened to the public but access to health care is selective based on financial resources in the absence of insurance plans and a social management system. Victims of motorcycle crash are admitted in a special 30 beds “bend skin” unit.

### Ethical considerations and patient consent

This study was approved by the institutional review board of the Faculty of Health Sciences of the University of Buea under number 2012-07-0054. Written consent to participate to the study was obtained either from the patient or a family member whenever possible. When this procedure could not be followed, a provisional administrative consent was requested from the hospital administration and patients who later on refused to participate were excluded from the cohort.

### Study participants and data collection

During the study period, all cases received in the emergency department of Laquintinie hospital after a traffic-related injury involving a motorcycle were identified. We included in the study all drivers (referred to in this study as riders) and pillion passengers of a motorcycle admitted in the emergency department for an injury sustained while they were involved in a motorcycle crash. All other cases of injury were excluded. Patients for whom basic epidemiological data such as age and sex could not be obtained, those who arrived dead, and those who did not consent were also excluded. Patients for whom inward admission was decided after initial management in the emergency department were followed-up in the corresponding ward for a maximum period of 7 days.

The data recorded included the following:

- Patient’s characteristics: epidemiological variables and profession.
- Crash characteristics: day and time of crash, type of collision, number of passengers on the motorcycle at the time of crash, position of the patient (rider or passenger), rider’s experience, helmet use and the notion of alcohol consumption within 6 h prior to the crash. For the purpose of description, we divided the days of a week to define week-days (from Monday to Thursday) and week-ends (from Friday to Sunday). We also divided the 24 h of a day as day time (from 6:00 am to 5:59 pm) and night (from 6:00 pm to 5:59 am). A commercial motorcyclist was defined as any person driving a motorcycle and transporting passengers against a payment.
- Lesions identified and recorded: Description of these lesion included identification of anatomical location, detailed description of the lesion, estimation of Glasgow Coma Scale and estimation of overall severity of injury using the Injury Severity Score (ISS).
- Outcome in the emergency department: discharge, inward admission, referral to another institution, or death in the emergency department or within 7 days following admission.

### Statistical analysis

Data were analysed using Epi-info® 2003. Student’s t test, chi-square or Fisher’s exact test were used to compare means and proportions. Results were considered significant for *p*-values less than 0.05.

### Reporting

The “Strengthening the Reporting of Observational Studies in Epidemiology” (STROBE) guidelines were used in reviewing and reporting the study [28].

## Results

### Patient’s characteristics

A total of 645 motorcycle crash victims were admitted in the emergency department. Twenty four (3.7 %) patients were excluded: four patients were declared dead on arrival, six did not have information on age and/sex available and fourteen declined to participate in the study. A total of 621 cases could finally be analyzed. These patients were involved in 405 motorcycle crashes.

The ages ranged from 4 to 85 years with a mean of 34.9 ± 16.8 years.

They were 325 males and 296 females, giving a sex-ratio of 1 · 1/1.

**Fig. 1:**
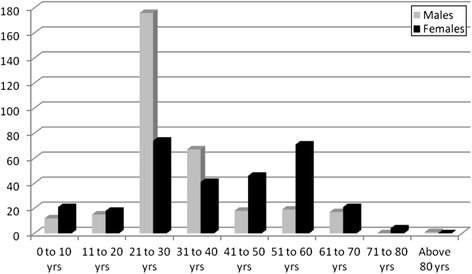
Distribution of motorcycle crash victims according to age and sex

As Fig. 1 show, the majority of patients (57.6 %) were aged 21–40 years. Patients aged between 41 and 60 years represented 25 % of the study population. Male sex significantly predominated in patients aged 21 – 40 years, while the female sex predominated in patients aged 41–60 years (*p* < 0.0001).

A total of 246 (39.6 %) patients were riders, all males. These included the 191 (77.6 %) commercial motorcyclists. The number of females who were pillion passengers (79 %) was significantly higher (*P* < 0.0001).

**Fig. 2:**
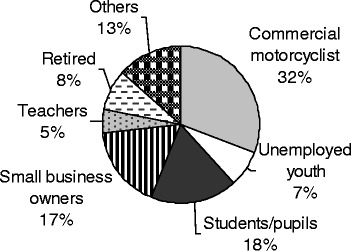
Distribution of motorcycle crash victims according to professional groups

Figure 2 shows the different professional categories involved in motorcycle crashes in Douala. Commercial motorcycle riders were the most affected professional group (*n* = 191).

### Crash characteristics

**Fig. 3:**
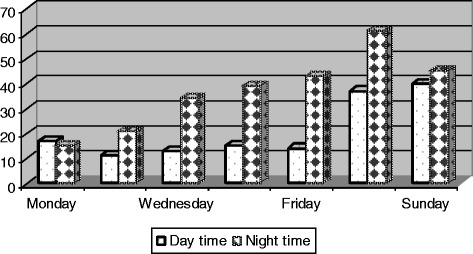
distribution of motorcycle crashes in Douala according to day and time

As Fig. 3 shows, a significant higher number (63.7 %) of crashes occurred over night (*p* < 0.0001). The risk of motorcycle crash was significantly higher on week-ends than on week-days (*p* < 0.0001).

Twenty two (9 %) of riders admitted alcohol consumption within 6 h prior to the crash. Half of the riders had more than 5 years experience.

Concerning helmet use, only 14 riders (5.7 %) and no passenger (0 %) had a helmet on at the time of the accident.

**Fig. 4:**
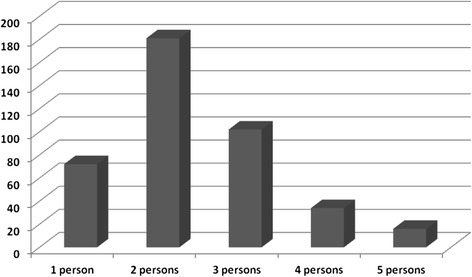
Number of persons present on the motorcycle at the time of the crash

As Fig. 4 shows, each motorcycle carried a mean 2.36 persons at the time of accident. A total of 147 (36.2 %) motorcycles carried 3 persons or more.

**Fig. 5:**
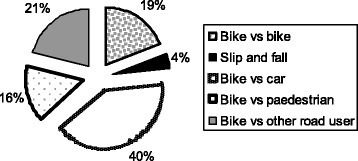
Distribution of our motorcycle crashes according to type of collision

Figure 5 indicates that collision between motorcycle and motor-vehicle (*n* = 162) or with another motorcycle (*n* = 77) represented 60 % of all crashes.

### Pattern of injuries

**Fig. 6:**
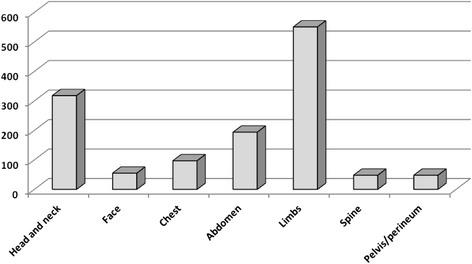
Distribution of motorcycle crash victims in Douala according to the anatomical region injured

A total of 1311 injuries were identified in our 621 victims giving a mean of 2.1 injuries per victim. As many as 429 (69 %) had more than one injury recorded. Limbs (*n* = 551), head and neck (*n* = 318), and abdomen (*n* = 194) were the most frequently injured anatomical regions (Fig. 6). Riders carried a higher risk of sustaining an injury to head and neck than pillion passengers (*p* < 0.0001). Riders and pillion passengers carried equal risk of injury to the lower limbs (*p* = 0.15).

As Table 1 shows, globally, soft tissue lesions represented almost 65 % of lesions recorded.

**Table 1:** Nature of lesion in victims of motorcycle crashes in Douala, Cameroon

Lesion	Number	Percentage
Abrasions/Bruises (defined as lesions not requiring suturing)	498	38
Lacerations (defined as lesions requiring suturing)	352	26.8
Traumatic brain injury	46	3.5
Bone fractures	299	22.9
Joint dislocations	7	0.5
Limb traumatic amputations	13	1
Haemothorax/Pneumothorax	8	0.6
Blunt injury to the abdomen	36	2.7
Penetrating abdominal injury	4	0.3
Urethral rupture	1	0.1
Injury to the anus	1	0.1
Spinal cord injury	10	0.8
Crush injury to a limb	36	2.7
Total	1311	100

The Glasgow coma scale (GCS) could be estimated in 588 patients. It ranged from 3 to 15 with a mean of 14 · 1. A total of 538 (91.5 %) patients had a GCS of 13 or more.

According to Table 2, more than 53 % of fractures were located in the lower limb and 21 % in the upper limb. The most commonly fractured site was the leg with 113 (37.8 %) of cases involving the tibia/fibula.

**Table 2:** Distribution by fracture site of victims who sustained a fracture in a study of motorcycle crash victims in Douala, Cameroon

Site	Number	Percentage
Skull	24	8
Spine	16 (6 cervical spine)	5 · 3
Maxillo-facial	17	5 · 7
Ribs	19	6 · 4
Upper limb		
Clavicle/scapula	2	0 · 7
Humerus	20	6 · 6
Radius/Ulna	29	9 · 7
Wrist and hand	12	4
Lower limb		
Pelvis girdle	11	3 · 7
Femur	19	6.4
Tibia/fibula	113	37 · 8
Foot	17	5 · 7
Total	299	100

ISS could be calculated precisely in 534 (86 %) patients. It ranged from 1 to 41 with a mean of 7.4 ± 9.6. It was 8.8 for riders and 5.9 for passengers. Mean ISS was significantly higher for riders than for pillion passengers (*p* < 0.001).

### Outcome

Analysis of the destination of patients after receiving treatment in the emergency department indicated that 418 (67.3 %) of patients were discharged back home, 126 (20.3 %) were admitted in one of the surgical wards or in the operation room and 38 (6.1 %) patients were admitted in the Intensive Care Unit. This gave an overall admission rate of 26.4 %. Referral to another centre concerned 12 (2 %) of patients. Twenty seven patients (4.3 %) died. Thirteen died in the emergency department and fourteen others died within seven days following admission. Mortality rate was significantly higher for riders (6.9 % fatality rate) than for passengers (2.7 % fatality rate), (*P* = 0.011). Mean GCS was significantly lower in those who died than in those who survived (*p* < 0.0001). Mean ISS (16.3) was significantly higher in those who died than in those who survived (*p* < 0.0001).

## Discussion

To the best of our knowledge, this is the first report of a cohort analysis exclusively devoted to the problem of motorcycle related injuries in an urban centre in Sub-Saharan Africa with regards to crash and patient’s characteristics and pattern of injuries.

Our findings indicate that motorcycle related injury victims consulting in Laquintinie Hospital can be divided into two main age groups with different sex-distribution: the classical group of patients aged 20 to 40 years with a majority of males and a second group of patients aged 41 to 60 years with a majority of females who are most often pillion passengers. As many as one third of crash victims develop a serious injury including head injuries and fractures. They generally get injured while circulating without a helmet over the week-end and in the night. The crash usually results from collisions with a motor-vehicle or another motorcycle. Motorcycles involved in crashes in Douala are often overloaded. Head and limb appear to be the most affected anatomical body areas. Death affects riders more than pillion passengers and usually occurs as a consequence of head involvement.

The main limitation of this study is the fact that it is not a community based study.

Some other concerns are likely to interfere with the generalizability of our results. The analysis of head involvement using GCS and the estimation of the overall severity of injury using ISS could be biased by the fact that these data were not available for all patients. However, these problems concerned a limited number of patients, generally less than 10 %.

The group of patients aged between 20 and 40 years is the typical age group of injury victims in general and motorcycle crash victims in particular as reported in most available studies [7, 19, 23, 25, 26, 29, 30]. But the second peak of incidence described in our report which concerns patients aged 41–60 years with a large majority of females, mostly pillion passengers is unusual. This feature probably accounts for the unprecedented sex-ratio of 1.1/1 described in our report. All existing studies report a male to female ratio ranging from 2.5/1 to 46.5/1 [11, 16, 17, 21–23, 25, 30, 31]. Many describe a massive male predominance above 95 % [11, 22, 31]. Fitzharris et al. also reported a very large majority of male riders, but only 30 % of their passengers were females [21].

These findings could be an indication of a greater involvement of females in income generating activities in Douala. This could also reflect a reduction in the perception of the risk of motorcycle crash in females aged above 40 years. Generally, African countries completely lack studies addressing the issue of perception of the risk of traffic related injuries of all types [32].

The analysis of crash characteristics confirms the vulnerability of motorcycles to other motorised road users [11, 16, 30, 33], especially in the night [21, 23] when activities are shifted in the most popular areas of the city. In a recent report from a country with similar characteristics, nearly 50 % of commercial motorcyclists admitted carrying two to four pillion passengers [34]. The high rate of collision between two motorcycles described in our report is rare in the literature. Also, our study identified neither injuries sustained in a collision between a motorcycle and a road obstacle such as a tree nor the classical “slide and fall” mechanism. This is probably explained by the fact that in Douala roads are designed for car use with no motorcycle bands and no obstacles. Cars and motorcycles then share the same driving space and this favours the car versus motorcycle collision.

Many previous reports describe the pattern of injuries specific to motorcycle crash victims and the vulnerability of head and lower limbs [11, 12, 15, 17, 22, 23, 31, 35], often in combination [25]. Generally, head involvement is responsible for or is a major contributor to death [12, 17, 22, 23, 33]. Limbs generally seem to be vulnerable to fractures, particularly the tibia and fibula [11, 25, 31, 36]. However, very few reports compare riders and pillion passengers in terms of anatomical location and severity of injuries [21, 24, 26]. Contrary to our description of a greater vulnerability of the head in riders, Fitzharris et al. reported no or few differences between riders and passengers in terms of anatomical location and severity of injury [21]. Zhao et al. rather report a greater vulnerability of hands, perineum, chest, and abdomen in riders [24]. This is probably related to a greater rate of helmet users in the areas where these other studies were conducted.

The low mean ISS score probably accounts for the low Intensive Care Unit admission rate and low mortality rate generally reported [13, 23, 31].

This hospital-based analysis can serve as a baseline for understanding the specific problem of motorcycle related injuries in the city of Douala. It is also an invitation for a more in-depth analysis which would ideally be community based or at least include most health institutions in Douala which are involved in the management of injury victims. This would help confirm some of our findings such as the identification of a new vulnerable group represented by females aged above 40 years. There is need to design a special message for these females who use commercial motorcycles as a mean of transportation to increase their awareness of the dangers of motorcycle use in the absence of protective measures.

The other findings, especially those related to crash characteristics are likely to reflect the general features of traffic crash involving motorcycles in other urban areas of Cameroon where the absence or the non-implementation of legal measures is the rule. They clearly indicate the need for action on a number of well known modifiable cost-effective risk factors of motorcycle related injuries such as helmet use and reflective coats in the night for both riders and passengers and the limitation of the number of pillion passengers. Long term measures such as redesigning the roads and rules of circulation to avoid mixing of incompatible users should also be considered.

## Conclusions

In the context of the high and increasing use of motorcycles for commercial purposes in Douala and urban settings in sub-Saharan Africa, the data provided by this study on the nature and severity of these injuries are likely to contribute to the conception and implementation of preventive measures as well as the planning of appropriate specific care to be provided to this category of injury patients. These findings confirm the growing problem of motorcycle injuries in LMICs and a strong recommendation towards the need of developing organized urban transports in Douala and other large cities in Cameroon. The findings of this report would also contribute in the design of national and international guidelines targeting motorcycle injuries and taking into account some of the crash characteristics highlighted in this study such as night collisions and the large number of females passengers. A more comprehensive approach starting from the site of accident and targeting head and limb involvement is more likely to reduce the burden of these injuries in terms of mortality and disability.
